# Tyrosine kinase inhibitors - balancing the haemostatic scales: a review of associated thrombosis and bleeding

**DOI:** 10.1007/s11239-025-03151-w

**Published:** 2025-09-15

**Authors:** Lloyd E. Butel-Simoes, Ammar Albayati, Jie Yu, Thomas Quirk, Shanathan Sritharan, Matthew French, Joshua D. Bennetts, Doan T. M. Ngo, Aaron L. Sverdlov

**Affiliations:** 1 Cardiovascular Department, John Hunter Hospital, Newcastle, NSW Australia; 2 Newcastle Centre of Excellence in Cardio-Oncology, The University of Newcastle, Hunter Medical Research Institute, Calvary Mater Newcastle, Newcastle, NSW Australia; 3 Hunter Medical Research Institute, Kookaburra Cct, New Lambton Heights, Newcastle, NSW 2305 Australia; 4 School of Medicine and Public Health, University of Newcastle, John Hunter Hospital, New Lambton Heights, NSW 2305 Australia; 5 School of Biomedical Sciences and Pharmacy, University of Newcastle, Callaghan, NSW 2308 Australia

**Keywords:** Tyrosine kinase inhibitors, Cancer, Bleeding, Thrombosis, Mechanisms

## Abstract

Tyrosine kinase inhibitors (TKIs) have revolutionised cancer therapy, significantly impacting survival and outcomes by targeting specific signalling pathways that are necessary for tumour survival. Despite their clinical efficacy, TKIs exhibit a complex toxicity profile. Many of the signalling pathways that are targeted by TKIs are shared with normal homeostatic processes, including those responsible for modulating thrombosis and bleeding. The risk profile of thrombosis and bleeding associated with TKIs varies considerably across agents. Multi-kinase inhibitors, particularly those targeting the breakpoint cluster regio-abelson murine leukaemia 1 gene mutation (BCR-ABL) (i.e., nilotinib and ponatinib), significantly elevate arterial thrombotic events. This thrombosis risk is driven by endothelial dysfunction, accelerated atherosclerosis, platelet hyper-reactivity, and impaired fibrinolysis. Similarly, vascular endothelial growth factor (VEGF) pathway inhibition contributes markedly to thrombotic vascular complications by reducing vasodilators like nitric oxide and promoting pro-thrombotic endothelial environments. TKIs targeting the VEGF receptor (VEGFR-TKIs) (i.e., sunitinib and regorafenib) and brutons tyrosine kinase (BTK) inhibitors (i.e., ibrutinib), increase bleeding risk through platelet dysfunction, thrombocytopenia, and interactions affecting coagulation pathways. Optimal management of these medications encompasses careful baseline cardiovascular and bleeding risk assessments, proactive modification of modifiable risk factors, and vigilant patient monitoring. Prophylactic antithrombotic therapy necessitates cautious individualised evaluation and comprehensive patient monitoring strategies. TKIs exemplify the advancements in precision oncology but necessitate nuanced management of their complex vascular toxicities. A multidisciplinary cardio-oncology approach involving detailed patient education, robust risk stratification, and collaborative clinical management is essential. Future research should aim to clarify TKI-specific haemostatic mechanisms and develop predictive biomarkers, enabling tailored therapeutic strategies to optimise clinical outcomes and reduce adverse events..

**Graphical Abstract:**

Mechanisms and management of tyrosine kinase inhibitor-associated thrombosis and bleeding. Tyrosine kinase inhibitor (TKI) therapy can target multiple oncogenic pathways. Despite their therapeutic efficacy, TKIs exert dual haemostatic off-target effects on vascular and platelet homeostasis, predisposing patients to both thrombosis and bleeding. This figure presents proposed mechanisms and management strategies for both complications. VEGFR, Vascular endothelial growth factor receptor; BCR-ABL, Breakpoint cluster region-Abelson; ALK, Anaplastic lymphoma kinase; FLT3, Fms-like tyrosine kinase 3; PDGFR, Platelet-derived growth factor receptor; EGFR, Epidermal growth factor receptor; JAK, Janus kinase; BTK, Bruton’s tyrosine kinase; NO, Nitrous oxide; PAI-1, Plasminogen activator inhibitor-1; IL-1β, Interleukin 1β; ROS, Reactive oxygen species; vWF, von Willebrand factor; GPVI, Platelet glycoprotein VI; NETs, Neutrophil extracellular traps; pMCS, Percutaneous mechanical circulatory support; FGFR, Fibroblast growth factor receptor; GP1b, Glycoprotein 1b; SSRIs, Selective serotonin reuptake inhibitors; CV, Cardiovascular; ECG, Electrocardiogram; BP; Blood pressure; DM, Diabetes mellitus; HTN, Hypertension; DOACs, Direct oral anticoagulants; MDT, Multidisciplinary team; GI, Gastrointestinal; NSAID, Non-steroidal anti-inflammatory drug (**Fig a).**

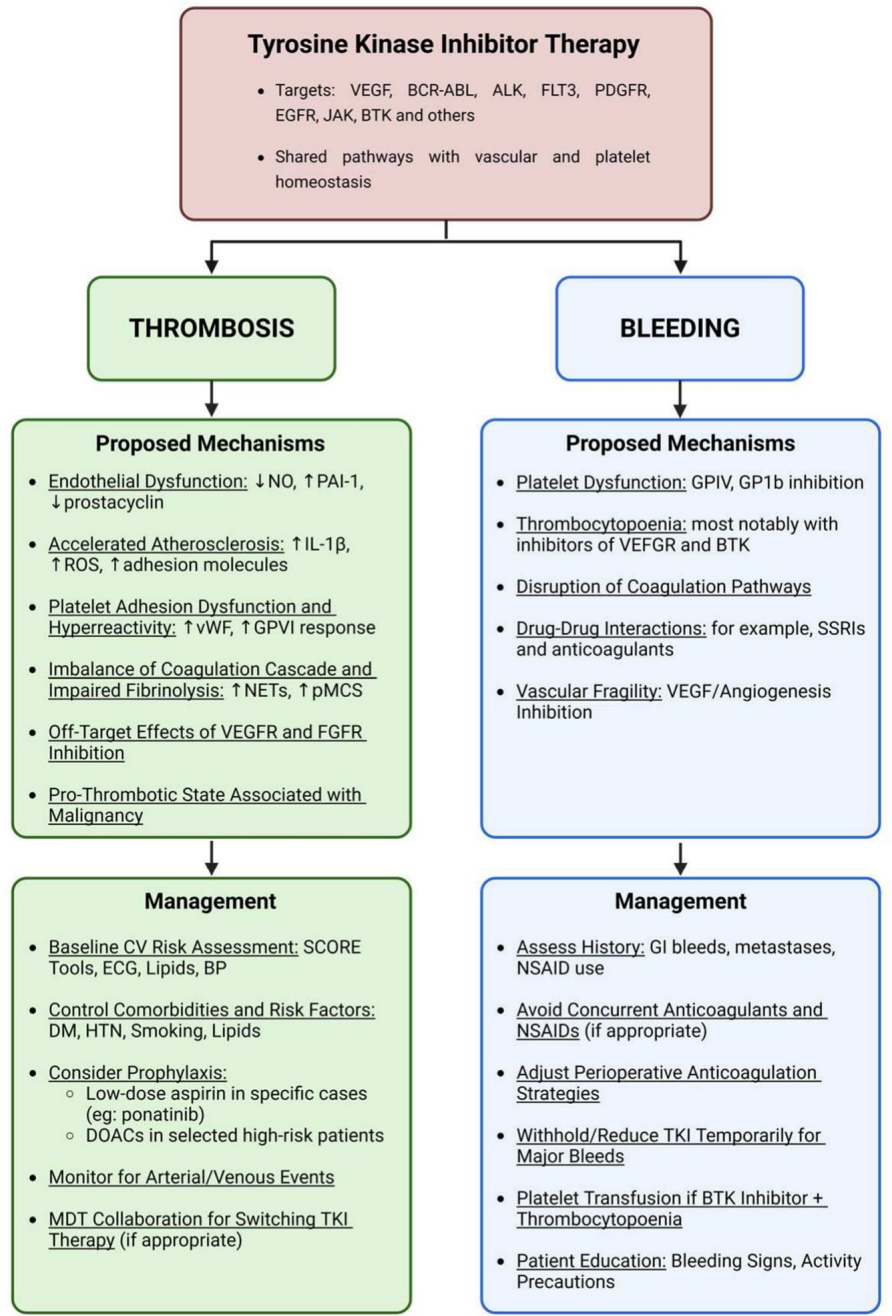

## Highlights

- Tyrosine kinase inhibitors (TKIs) disrupt haemostatic balance, contributing to agent-specific risks of both thrombosis and bleeding via diverse on- and off-target effects on endothelial function, platelets, and coagulation pathways.
- Thrombotic complications—particularly arterial events—are most frequently associated with VEGFR and BCR-ABL-targeting TKIs and are driven by endothelial injury, platelet activation, and accelerated atherogenesis.
- Bleeding risk is highest with BTK inhibitors due to impaired platelet aggregation, though newer agents with increased selectivity show reduced haemorrhagic toxicity, reflecting the importance of kinase selectivity.
- Clinical decision-making must navigate a narrow therapeutic window, especially in patients requiring antiplatelet or anticoagulant therapy, calling for multidisciplinary collaboration and individualised risk-benefit assessment in cardio-oncology settings.
- Implications for future directions: Mechanism-based risk stratification, integration of platelet function and endothelial biomarkers, and rational design of TKIs with improved haemostatic safety profiles are key priorities for mitigating therapy-induced vascular complications.

## Introduction

Targeted therapies are now a cornerstone of modern cancer therapy and can be found across almost all types of cancer treatment. Significant progress has been made with precision targeting of the protein tyrosine kinases in cancer cells, which plays a pivotal role in promoting cancer cell growth and malignant proliferation. Targeting and inhibition of the receptor of specific subgroups of the tyrosine kinase can therefore halt growth and alter the course of cancer progression [1].

The tyrosine kinase family has grown from our early knowledge of foundational therapies, such as Imatinib which was approved in 2001 for the treatment of Ph+ chronic myelogenous leukaemia, to what now encompasses a “super-family” of new and emerging TKIs that target various sites and configurations of the tyrosine kinase receptor and downstream enzymatic pathways. There are currently 80 FDA approved small molecule protein-kinase inhibitors available, and over 400 registered clinical trials ongoing worldwide, with rapid progression in the field of precision therapies not only across cancer but also in many areas including autoimmune immunological, rheumatological and even cardiovascular disease treatments [2, 3]. Given the heterogeneity of such a broad class of protein-kinase targets, it would be expected that both treatment and adverse effects related to treatment also vary considerably between subgroups of TKIs.

Cardiologists are increasingly involved in the care of cancer patients (the emerging field of “Cardio-Oncology”) to help manage TKI-related hypertension, arrhythmias, and vascular toxicities. Notably, some highly effective TKIs have shown unexpected rates of arterial thrombosis, influencing how clinicians select and monitor therapy [4]. On the other hand, certain TKIs can impair platelet function or cause cytopenia’s, predisposing patients to bleeding [4].

This scoping review will focus on the aspects of thrombosis, bleeding and platelet dysfunction associated within the TKI classes, and draw attention to established and emerging subclasses within the TKI superfamily.

### What are tyrosine kinase inhibitors and how do they work

Tyrosine kinases are enzymes that catalyse the transfer of a phosphate group from adenosine triphosphate (ATP) to the tyrosine residues of specific substrate proteins, a process known as phosphorylation. This post-translational modification plays a pivotal role in regulating various cellular processes including growth, proliferation, differentiation, metabolism, migration and apoptosis [5]. Dysregulation of tyrosine kinase activity, often due to mutations or overexpression, has been implicated in the pathogenesis of numerous cancers [5, 6].

Tyrosine kinases are broadly classified into two main categories: receptor tyrosine kinases and non-receptor (cytoplasmic) tyrosine kinases. TKIs are transmembrane proteins that, upon ligand binding, undergo dimerization and autophosphorylation to initiate intracellular signalling cascades. Non-receptor tyrosine kinases, lacking transmembrane domains, are located in the cytoplasm and typically associate with other cellular receptors to propagate signalling pathways [7, 8].

Despite their selectivity, many TKIs effect multiple kinases. For example, sunitinib was designed to inhibit VEGFr but also blocks platelet derived growth factor receptor (PDGFR) and proto-oncogene c-KIT (c-KIT), and ponatinib (a third-generation BCR-ABL inhibitor) has potent activity not only against BCR-ABL but also all VEGF receptor subtypes and fibroblast growth factor receptor (FGFR) [8, 9]. This multi-kinase inhibition can enhance anti-tumour efficacy (especially in tumours that rely on multiple growth pathways), but also increases the risk of off-target toxicities [10].

TKIs are therapeutic agents designed to interfere with the aberrant signalling pathways mediated by these enzymes. They function by competitively binding to the ATP-binding site or allosteric sites of the kinase, thereby preventing phosphorylation of target proteins and subsequent signal transduction. This inhibition can lead to reduced tumour growth and angiogenesis, as well as the induction of cancer cell apoptosis [1, 6, 7].

**Fig. 1:**
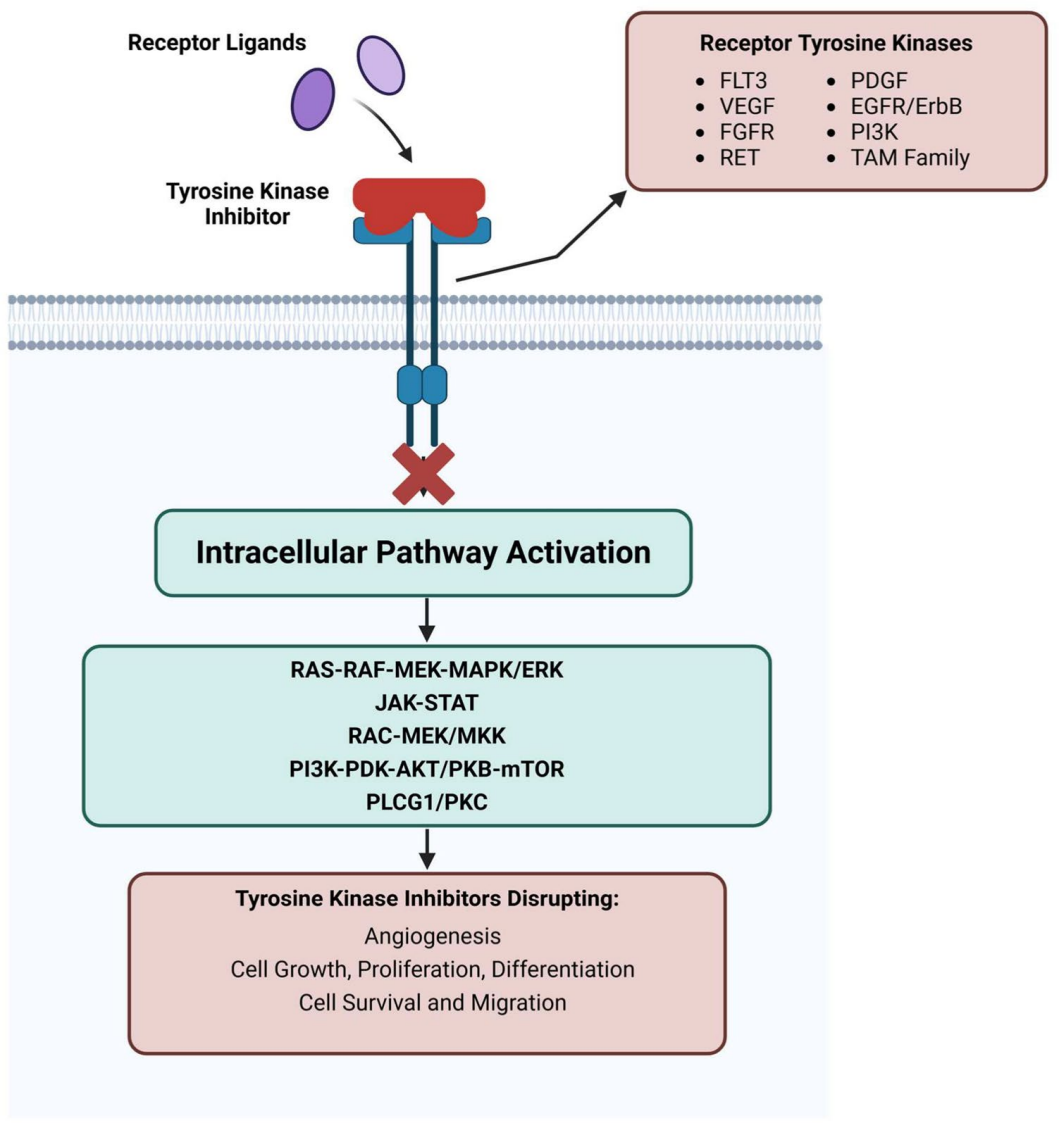
Downstream biological effects of tyrosine kinase inhibitors. Cell membranes express several major tyrosine kinase families. Binding of receptor ligands to tyrosine kinases activates different intracellular pathway cascades, causing downstream biological effects. Tyrosine kinase inhibitors can disrupt these biological mechanisms, leading to disruptions in cell growth and survival. Receptor Tyrosine kinases (RTKs), Fms-like tyrosine kinase 3 (FLT3), Vascular endothelial growth factor receptor (VEGFR), Fibroblast growth factor receptor (FGFR), REarranged during transfection (RET), Platelet-derived growth factor (PDGF), Epidermal growth factor receptor (EGFR), Erythroblastic leukemia viral oncogene homolog (ErbB), Phosphoinositide 3-kinase (PI3K), Tyro3, Axl, and Mer Receptors (TAM), Rat sarcoma virus oncogene (RAS), Rapidly accelerated fibrosarcoma (RAF), Mitogen-activated protein kinase kinase (MEK), Mitogen-activated protein kinase (MAPK), Extracellular signal-regulated kinase (ERK), Janus kinase-signal transducer and activator of transcription (JAK-STAT), Ras-related C3 botulinum toxin substrate (RAC), Mitogen-activated protein kinase kinase (MEK), Mitogen-activated protein kinase kinase (MKK), 3-Phosphoinositide-dependent protein kinase-1 (PDK), Protein kinase B (AKT), Protein kinase B (PKB), Mechanistic target of rapamycin (MTOR), Phospholipase C gamma 1 (PLCG1), Protein kinase C (PKC)

TKIs can be classified into distinct types; Type I bind the active DFG-in conformation of the kinase, while Type II bind and stabilize the inactive DFG-out conformation. Both Type I and II are both competitive inhibitors binding the ATP-binding pocket and other selective domains of the kinase receptor not allowing for activation. Type III and Type IV inhibitors vary in that they are allosteric inhibitors of the tyrosine kinase with variable binding sites such as mitogen-activated protein kinase kinase (MEK) inhibitors (type III) and Asciminib (type IV) [1, 8]. TKI can also be classified by their respective subfamilies as demonstrated in the Table 1 (adapted from Tomuleasa et al. and Roskoski [3, 8]) and their mechanisms of action depicted in Fig. 1.

**Table 1:** Currently approved TKIs, approval date, indication, target and the reported thrombosis and bleeding risk

Tyrosine kinase inhibitor	Approval date	Indication(s)	Thrombosis risk	Bleeding risk	Target
Imatinib (Gleevec)	2001	CML, Ph+ ALL, GIST	Low risk of thrombosis, but bleeding events and anaemia can occur	Risk of gastrointestinal bleeding, intestinal perforation, and intratumoral haemorrhage	BCR-ABL, KIT, PDGFRA
Dasatinib (Sprycel)	2006	CML, Ph+ ALL	Associated with vascular events, including thrombosis (low risk)	Increased bleeding (~23% incidence), primarily gastrointestinal; rare severe CNS haemorrhage[11]	BCR-ABL, SRC family kinases
Nilotinib (Tasigna)	2007	CML (resistant/intolerant)	Increased risk of arterial occlusive events in 3–4% by 3 years and up to 13% over 5y [12]	Thrombocytopenia-related bleeding; infrequent significant haemorrhagic events reported	BCR-ABL
Ponatinib (Iclusig)	2012	CML, Ph+ ALL	Serious arterial thrombosis in ~20% of patients over 5 years [13]. EPIC trial stopped early due to 7% arterial events in 5 months [14]. High thrombosis risk (>25%)[15]	Rare serious haemorrhages, typically in context of severe thrombocytopenia [16]	BCR-ABL, KIT, PDGFR
Bosutinib (Bosulif)	2012	CML (resistant/intolerant)	1–2% risk of thrombosis, particularly arterial [17]	Bleeding associated primarily with thrombocytopenia; significant haemorrhagic events uncommon [18, 19]	BCR-ABL, SRC family kinases
Erlotinib (Tarceva)	2004	NSCLC, Pancreatic cancer	Low thrombosis risk, but rare thrombotic events in pre-existing conditions [17]	Gastrointestinal bleeding, increased risk when combined with NSAIDs, steroids, or anticoagulants	EGFR
Gefitinib (Iressa)	2003	NSCLC (EGFR mutated)	Rare thrombosis, more common in patients with pre-existing conditions	Uncommon bleeding events: rare gastrointestinal perforation or cerebral haemorrhage reported	EGFR
Afatinib (Gilotrif)	2013	NSCLC	No strong association	Rare gastrointestinal perforation or haemorrhage reported	EGFR, HER2, ErbB4
Osimertinib (Tagrisso)	2015	NSCLC	No strong association	Rare severe bleeding events, including gastrointestinal and pulmonary haemorrhages; thrombocytopenia-related bleeding may occur	EGFR
Dacomitinib (Vizimpro)	2018	EGFR-mutant NSCLC	No strong association	No significant direct bleeding risks reported; potential indirect increase in bleeding risk due to interaction with anticoagulants via P-glycoprotein (P-gp) and breast cancer resistance protein (BCRP) inhibition	EGFR
Lapatinib (Tykerb)	2007	HER2+ breast cancer	No strong association	Minimal direct bleeding risk: may cause thrombocytopenia (especially in combination with capecitabine) and hepatotoxicity, indirectly increasing bleeding potential. Rare epistaxis reported	HER2, EGFR
Neratinib (Nerlynx)	2017	HER2+ breast cancer	No strong association	Rare epistaxis reported; potential hepatotoxicity may indirectly lead to bleeding or bruising	HER2
Tucatinib (Tukysa)	2020	HER2+ breast cancer	No strong association	May cause thrombocytopenia, leading to increased risk of bruising, bleeding gums, and epistaxis. potential increased bleeding risk with concomitant use of NSAIDs or anticoagulants	HER2
Crizotinib (Xalkori)	2011	NSCLC (ALK mutations)	Low thrombosis risk; rare cases of pulmonary embolism	Risk of thrombocytopenia-related bleeding; potential for unusual bruising or bleeding events (e.g., epistaxis)	ALK, ROS1
Ceritinib (Zykadia)	2014	NSCLC (ALK mutations)	Low thrombosis risk, more common with liver toxicity	Potential for increased bleeding risk due to interactions with anticoagulants, as ceritinib inhibits P-glycoprotein (P-gp) and breast cancer resistance protein (BCRP), leading to elevated levels of DOACs. Additionally, ceritinib may cause hepatotoxicity, which can manifest as easy bruising or bleeding [20]	ALK
Alectinib (Alecensa)	2017	ALK + NSCLC	No strong association	Minimal bleeding risk reported	ALK, RET
Brigatinib (Alunbrig)	2017	ALK + NSCLC	No strong association	Minimal bleeding risk reported	ALK
Lorlatinib (Lorbrena)	2018	ALK + NSCLC	No strong association	Minimal bleeding risk reported	ALK
Axitinib (Inlyta)	2012	RCC	2–3% risk of venous thromboembolism (VTE) [21]	May increase bleeding risk, especially when combined with anticoagulants	VEGFR-1, 2, 3, PDGFR
Regorafenib (Stivarga)	2012	CRC, GIST, HCC	Associated with VTE (1.4–1.6% incidence) [22]	Up to 8.6% of all grade haemorrhage incidence [22]. 1.2% serious bleeding	VEGFR, KIT, PDGFR
Cabozantinib (Cabometyx)	2016	RCC, HCC, Medullary thyroid cancer	Moderate thrombosis risk (VTE and arterial)	May increase bleeding risk, particularly in patients with prior hemorrhage history	VEGFR, MET, AXL, KIT
Pazopanib (Votrient)	2009	RCC, Soft Tissue Sarcomas	VTE incidence ~3% amongst VEGFR-TKIs; no significant increase vs non-TKI therapy (RR ~0.91, *p* = 0.64) [23]	Associated with hemorrhagic events in ~22% of patients; includes serious and fatal bleeding such as gastrointestinal, intracranial, and pulmonary hemorrhage Novartis Pharmaceuticals. (2024). *Votrient (pazopanib) prescribing information*. Retrieved from https://www.novartis.com/us-en/sites/novartis_us/files/votrient.pdf [24]	VEGFR
Sorafenib (Nexavar)	2005	HCC, RCC, DTC	ATE = 3–6% [25] VTE2-5% [23] Sorafenib and sunitinib Associated with approximate threefold increased relative risk of arterial thromboembolism [25]	Up to 15% of all grade haemorrhages and 9% serious bleeding [26]	VEGF, BRAF, KIT, FLT-3, RET
Palbociclib (Ibrance)	2015	HR+ breast cancer	Low thrombosis risk; rare with comorbidities	Minimal bleeding risk reported	CDK4, CDK6
Ruxolitinib (Jakafi)	2011	Myelofibrosis, Polycythemia vera	Increased thrombosis risk in myelofibrosis patients	Minimal bleeding risk reported	JAK1, JAK2
Lenvatinib (Lenvima)	2015	HCC, RCC, Endometrial carcinoma	Low to moderate arterial thrombosis risk	Associated with increased risk of bleeding, including serious and fatal events such as gastrointestinal and intracranial hemorrhage. Risk is elevated in patients with tumor invasion into major blood vessels [27]	VEGFR-1, 2, 3, FGFR, PDGFR
Vandetanib (Caprelsa)	2011	Medullary thyroid cancer	Low thrombosis risk; arterial events in some cases	Minimal bleeding risk reported	VEGFR, EGFR, RET
Sunitinib (Sutent)	2006	RCC, GIST, Pancreatic neuroendocrine tumors	Thrombosis risk (2–3%) including arterial events [28] Sorafenib and sunitinib Associated with approximate threefold increased relative risk of arterial thromboembolism [25]	Associated with increased risk of bleeding, including serious and fatal hemorrhagic events (e.g., gastrointestinal, pulmonary, and intracerebral hemorrhage) [24]	VEGFR-1, 2, 3, PDGFR, KIT
Toceranib (Palladia)	2009	Canine cancers	Thrombosis risk not well-studied in humans	Minimal bleeding risk reported	VEGFR, KIT
Tivozanib (Fotivda)	2017	RCC	Low thrombosis risk; some vascular events in comorbid patients	Minimal bleeding risk reported	VEGFR-1, 2, 3
Bortezomib (Velcade)	2003	Multiple myeloma, Mantle cell lymphoma	Low thrombosis risk; associated with thrombocytopenia and bleeding	Minimal bleeding risk reported	Proteasome
Baricitinib (Olumiant)	2018	Rheumatoid Arthritis	No strong association	Minimal bleeding risk reported	JAK1, JAK2
Upadacitinib (Rinvoq)	2019	Rheumatoid Arthritis	No strong association	Minimal bleeding risk reported	JAK1
Blinatumomab (Blincyto)	2014	Ph+ ALL	Thrombosis is rare but can occur with infection or cytokine release syndrome	Minimal bleeding risk reported	CD3, CD19
Olaparib (Lynparza)	2014	Ovarian cancer, BRCA mutations	Thrombosis risk is low, but combination with other treatments may increase risk	Minimal bleeding risk reported	PARP
Deucravacitinib (Tavalisse)	2022	Plaque psoriasis	Low to moderate thrombosis risk	Minimal bleeding risk reported	TYK2
Avapritinib (Ayvakit)	2020	GIST (PDGFRA mutations)	Low to moderate thrombosis risk	Minimal bleeding risk reported	KIT, PDGFRA
Idelalisib (Zydelig)	2014	CLL, FL, SLL	No strong association	Minimal bleeding risk reported	PI3Kδ
Acalabrutinib (Calquence)	2017	MCL, CLL/SLL, WM	No strong association	Bleeding events common (26–43%); serious hemorrhage (Grade ≥3) reported in 1–5%, generally lower risk compared to ibrutinib[29]	BTK
Zanubrutinib (Brukinsa)	2019	MCL, CLL/SLL, WM	No strong association	Bleeding events common; serious (Grade ≥3) hemorrhages in ~2% of patients, including intracranial and gastrointestinal bleeding [30]	BTK
Ibrutinib (Imbruvica)	2013	CLL, MCL, MZL	Arterial events: atrial fibrillation in ~10% (cumulative incidence 5.9% at 6 mo, 10.3% at 2 yrs)​, can lead to cardioembolism if not anticoagulated. No major increase in MI/stroke seen, but case reports exist [31]	Common bleeding events (~39%); major hemorrhage in ~4%, including rare fatal intracranial or gastrointestinal bleeding [32, 33]	BTK

### Mechanisms of thrombosis with tyrosine kinase inhibitors

Cancer patients are at a high baseline risk for both arterial and venous thromboembolism compared to the general population [34]. Malignancies such as pancreatic cancer, brain cancer, ovarian cancer and multiple myeloma have been shown to be particularly high-risk for inducing pro-thrombotic states [35]. Other risk factors for thrombosis may include age, metastatic burden, thrombocytosis, sepsis, hypoalbuminemia and congestive cardiac failure [36]_._ This risk may be further mediated through choice of treatment regimen, which is especially true for TKIs as they have been associated with a high rate of prothrombotic vascular events [10].

TKIs have been linked to thrombotic events in multiple clinical settings, ranging from arterial thromboses (myocardial infarction, ischemic stroke, peripheral arterial occlusion) to venous thromboembolism (deep vein thrombosis and pulmonary embolism). The prothrombotic effects appear to vary by TKI agent and target [10]. There are significant challenges when attributing vascular thrombotic risk to TKI therapy given the established and complex thrombotic risk in cancer patients [10].

TKIs are expressed in both tumour and non-tumour cells, resulting in the tissue agnostic effects of TKI therapies [10]. The mechanism of thrombosis is not completely understood; however, mechanisms may broadly be categorised into: endothelial dysfunction, atherosclerotic plaque progression, imbalance between coagulation cascade activation and reduced fibrinolysis and platelet dysfunction and platelet adhesion dysfunction [10, 37, 38].

It is important to recognise that the combination and complex interplay of all these factors accounts for thrombotic risk. For example cancer/TKI mediated endothelial dysfunction causes the release of pro-thrombotic factors and acceleration of atherosclerotic plaque formation, increases activation of the coagulation cascade along with impaired fibrinolysis, and can promote thrombo-inflammatory interactions (e.g., platelet-leukocyte aggregates and neutrophil extracellular traps) and altered action of platelet glycoprotein VI and fibrinogen [39].

Newer generation TKIs with low selectivity (dasatinib, ponatinib, nilotinib) are designed for greater potency against BCR-ABL, as well as for increased efficacy in patients with multi-drug-resistant mutations. Due to their multi-kinase effects, these drugs may be associated with an increased risk of thrombotic events, particularly due to vascular endothelial growth factor (VEGF) inhibition [40]. The VEGF pathway plays a major role in inhibiting angiogenesis within tumour cells, and is also responsible for endothelial cell regulation. The interruption of this pathway by TKIs may alter systemic coagulation processes and thus be responsible for the hypercoagulable state seen in these patients [25].

Some of our earliest understanding of how cancer treatment can promote thrombosis risk and vascular complications have come from studies of bevacizumab (VEGF inhibitor), which demonstrated that increased expression of Plasminogen activator inhibitor (PAI-1) on various cells and tissues (including platelets and thrombi) was potentially dysregulating coagulation and endothelial health [41, 42]. Furthermore, VEGF-TKI pathway disruption leads to reduced production of vasodilators and antithrombotic factors (like nitric oxide and prostacyclin), and can cause hypertension and vascular damage that further predisposes the patient to adverse thrombotic vascular events [43–45]. This risk was clinically highlighted in the results of a meta-analysis of several large VEGF inhibitor trials, which found an almost threefold increase in arterial thrombotic events compared with control subjects [25]. This pro-thrombotic risk with VEGF inhibitor therapies has been acknowledged by the FDA for many approved TKIs, including axitinib, regorafenib, Lenvatinib [46].

Accelerated atherosclerotic plaque generation and instability have been implicated as other important factors for pro-thrombotic states associated with some TKI therapies. The mechanism for this is not well delineated in the literature, however, it is believed that innate interleukin 1 beta(IL-1B) and IL-6-driven immune responses and increased expression of endothelial cell adhesion molecules are the primary drivers of atherosclerotic growth and plaque instability [10, 47]. Worsening hyperglycaemia and dyslipidaemia have also been implicated with some selective TKI treatments (Nilotinib), along with the increased production of reactive oxygen species which act as pro inflammatory and pro-atherosclerotic mediators (Nilotinib and Ponatinib) [48–50]. Similarly, VEGF inhibition causing decreased nitric oxide and increased mitochondrial superoxide generation can cause accelerated atherosclerotic progression. This has been demonstrated in murine models [51, 52].

TKIs can induce the inflammatory cell interactions and induce formation of neutrophil extracellular traps (NETs), promoting platelet-leukocyte complexes (PMCs) according to proposed models. Both NETs and PMCs are known to fuel thrombosis by providing a scaffold for clot formation and amplifying coagulation [53].

Another contributing factor to TKI-associated thrombosis is altered platelet activation and aggregation. Although TKIs vary in their effects on platelets (discussed further in the bleeding section), some TKIs may increase platelet reactivity and thereby foster thrombosis. Preclinical studies found ponatinib-treated mice had shorter bleeding times and hyperactive platelets, with enhanced platelet aggregation responses to stimuli (glycoprotein VI, a-thrombin and c-reactive protein) [50]. Endothelial cells under TKI exposure may release prothrombotic mediators, such as von Willebrand factor (vWF) from Weibel-Palade bodies, thereby increasing platelet adhesion. This was demonstrated in In-vivo models of ponatinib treated mice. This suggests TKIs can trigger endothelial cells to adopt a pro-adhesive, thrombogenic surface [54]. This paradoxical pro-platelet effect (despite ponatinib being a kinase inhibitor) likely arises from complex signalling crosstalk and endothelial interactions. In contrast, other TKIs like Dasatinib inhibit platelet function (see later), highlighting that platelet-mediated thrombotic risk is agent-specific [10].

## Clinical evidence of thrombotic risk associated with tyrosine kinase inhibitors

### BCR-ABL TKI therapies for chronic myeloid leukemia (CML)

Imatinib is a first generation BCR-ABL TKI used in the treatment of chronic myeloid leukaemia (CML). It has been well studied for almost two decades and has showed minimal vascular risk. However, studies of both second and third generation agents have demonstrated significantly higher thrombotic events. In the Evaluating Nilotinib Efficacy and Safety in Clinical Trials—Newly Diagnosed Patients (ENESTnd) trial comparing nilotinib to imatinib for newly diagnosed CML, cardiovascular events (coronary, cerebrovascular, or peripheral arterial) were markedly more frequent with nilotinib. At 3 years, arterial event rates were ~3–4% with nilotinib versus 2.1% with imatinib, and by 6 years the rates increased to 7.5% and 13.4% in the nilotinib 300 mg BID and 400 mg BID arms, respectively (versus 2.1% on imatinib) [55]. These late-emerging arterial occlusions with nilotinib often involved severe peripheral arterial disease requiring revascularization and are considered due to both thrombotic events and worsening of atherosclerotic disease [56, 57].

Ponatinib, a potent pan-kinase inhibitor, has an even more striking thrombotic profile as was demonstrated in the Ponatinib Ph-positive acute lymphoblastic leukemia [ALL] and CML Evaluation (PACE) trial for refractory CML. Ponatinib led to serious arterial thrombotic events in ~20% of patients over 5-year follow-up. In part, this is believed to be due to off target effects of treatment inhibition of VEGF and FGF receptor pathways [58]. These included cardiovascular (10%), cerebrovascular (7%), and peripheral arterial events (8%) [13].

The risk was dose-dependent, prompting dose reduction strategies and a “black box” FDA warning along with Ponatinib now more frequently considered a second line treatment strategy [10]. A subsequent front-line trial found similar compelling results when comparing ponatinib to imatinib and was terminated early (after ~5 months) when 7% of ponatinib treated patients experienced arterial occlusive events, majority of these where deemed severe [14].

Notably, other CML TKIs like dasatinib and bosutinib have not shown such high rates of arterial thrombosis; the risk seems particularly elevated with nilotinib and ponatinib, possibly related to their multi-kinase activity and metabolic effects.

### Angiogenic TKIs

Another group of TKIs which have raised similar concerns of thrombosis have been those targeting VEGFrs in solid tumours (e.g., sunitinib, sorafenib, pazopanib, axitinib). The mechanism of arterial thromboembolism is considered to be likely via endothelial effects and hypertension, as opposed to progression of atherosclerotic disease [25, 44]. A meta-analysis of trials including over 10,000 patients assessed VEGF-pathway targeted agents (Sunitinib and Sorafenib) and found a roughly threefold increased relative risk of arterial thrombotic events compared to controls [25]. In that analysis, the overall incidence of arterial events (such as myocardial infarction or stroke) was still relatively modest (in the order of a few percent), but clearly higher than in placebo/controls [25]. Similarly, two other large meta-analysis of various VEGF inhibitors (VEGFi) reported a modest range (2.8–3.5) relative risk increase of myocardial ischemia amongst treated patients [59, 60].

The FDA has also documented arterial thromboses with other VEGF/PDGF inhibitors and related agents such as pazopanib, regorafenib, lenvatinib, and even some endothelial growth factor receptor (EGFR) TKIs and monoclonal antibodies [46, 61].

Thus, any agent impairing angiogenesis signals can potentially precipitate arterial ischemic events, although the absolute risk for an individual patient remains fairly low (generally <5%). Risk factors (like pre-existing cardiovascular disease) greatly modulate the absolute risk.

### Non-angiogenic TKIs and others

Some newer targeted TKIs have less evidence of thrombosis, but isolated signals exist. BTK inhibitors (e.g., ibrutinib for B-cell malignancies) are primarily associated with bleeding (see next section) rather than thrombosis; however, they do predispose patients to atrial fibrillation in up to 10–16% of cases. This indirectly increases stroke risk if not managed [62].

Anaplastic lymphoma kinase (ALK) inhibitors (crizotinib, alectinib) and other modern TKIs have not shown strong thrombosis signals in trials, aside from events attributable to the cancer itself [10].

It is important to distinguish arterial vs. venous risk. Interestingly, TKIs that markedly increase arterial thromboses do not always confer a similar increase in venous clots. The VEGFR inhibitor class, for instance, shows a clear arterial risk, but a more equivocal effect on venous thromboembolism (VTE). A 2013 meta-analysis of >4400 patients on VEGFR-TKIs (sunitinib, sorafenib, pazopanib, vandetanib) found an overall VTE incidence of ~3% on these TKIs, and *no statistically significant increase* in VTE risk compared to patients on non-TKI therapy [23]. The relative risk of VTE with VEGFR-TKIs was ~0.91, found to be essentially neutral [63]. This suggests that cancer type and patient factors (e.g., immobility, pro-coagulant tumours) are the dominant drivers of VTE, rather than the TKI itself. In contrast, arterial events seem more directly attributable to certain TKIs’ pharmacologic effects on vessels and platelets [64]. The key point is that not all TKIs confer the same thrombotic risk profile.

In summary, each TKI (and class of TKIs) has a distinct thrombotic “fingerprint”. Clinicians should familiarize themselves with the specific risks of the TKI being prescribed. For example, a patient on ponatinib needs aggressive cardiovascular risk factor management and monitoring for arterial ischemia, whereas a patient on ibrutinib needs monitoring for atrial fibrillation and might actually have a reduced platelet aggregation tendency. Understanding these nuances allows for personalized preventative strategies and prompt recognition of thrombosis if it occurs.

### Bleeding and platelet inhibition associated with TKIs

Targeted tyrosine kinase inhibitors (TKIs) have improved efficacy over conventional chemotherapy [65]. However, these agents are associated with significant adverse effects in several trials and case reports, including an increased risk of bleeding and platelet inhibition [30, 51]. A better understanding of the mechanisms underlying bleeding and platelet dysfunction is crucial to develop a personalised method to improve patient outcomes [66, 67].

VEGFR-TKIs have improved outcomes for certain types of solid tumours [68]. However, one of the common side effects of VEGFR-TKIs is increased risks of bleeding and platelet aggregation inhibition as well as thrombocytopenia [69–72].

A meta-analysis of 23 trials that included 6779 patients reported that 708 out of 4934 patients (16.7%, 95% CI 12.7–21.5%) treated with VEGFR-TKIs had all grades of bleeding events, whilst high-grade bleeding events (defined as ≥grade 3, based on the National Cancer Institute’s Common Terminology Criteria for Adverse Events) occurred in 100 out of 6597 patients This represents an incidence rate of 2.4% (1.6–3.9%) [73]. According to data from phase III trials, the use of TKIs (sunitinib and sorafenib) presented a twofold increase in the risk of all-grade bleeding events (95% CI, 1.14–3.49; *p* = 0.015) [73]. There was no statistical significance observed between renal cell cancers and non-renal cancers [73].

Another meta-analysis of 27 randomised controlled trials demonstrated that the use of VEGFR-TKIs was consistently associated with increased haemorrhagic events by 67% (RR 1.67, 95% CI 1.19–2.33, *p* = 0.003) [74]. Though the overall incidence of all grade and high-grade haemorrhagic events was lower at 9.1 and 1.3%, respectively [74].

Furthermore, a broader network meta-analysis included 50 RCTs of 16,753 cancer patients and analysed bleeding profiles for eleven U.S. FDA approved VEGFR-TKIs (Apatinib, Brivanib, Cabozantinib, Lenvatinib, Motesanib, Nintedanib, Pazopanib, Regorafenib, Sorafenib, Sunitinib and Vandetanib) [75]. Looking at all-grade bleeding events, 1283 of 9575 patients receiving VEGFR-TKIs were at an increased bleeding risk compared to 617 of 7178 patients in the control group [75]. The risk of high-grade bleeding was not significantly increased in VEGFR-TKIs group (RR 1.22; 95% CI 0.87–1.71, *p* = 0.74)[75]. Interestingly, sunitinib (Odds Ratio, OR = 3.31, 95% CI 2.34–4.69) and regorafenib (OR = 2.92, 95% CI 1.50–5.71) showed a significantly higher risk of haemorrhagic events compared to placebo [75].

Cancer induces a hypercoagulable state [76, 77]. It is critical to balance the benefit and risk between bleeding and thrombosis. Low-molecular-weight heparin (LMWH), an indirect factor Xa inhibitor, has been the standard treatment for cancer-associated thrombosis for the past decade [77–80]. Clinical trials, including the Randomized Comparison of Low-Molecular-Weight Heparin versus Oral Anticoagulant Therapy for the Prevention of Recurrent Venous Thromboembolism in Patients with Cancer (CLOT) [81] and Comparison of Acute Treatments in Cancer Haemostasis (CATCH) trial [82], have demonstrated its efficacy in preventing recurrent venous thromboembolism in cancer patients. However, LMWH carries an increase in major bleeding risk ranging from 2.7 [82] to 6% [81].

A retrospective study of 86 patients with cancer receiving VEGFR-TKIs (pazopanib, sunitinib, sorafenib, axitinib, regorafenib, vandetanib, lenvatinib, or cabozantinib) and factor Xa inhibitors (LMWH or DOACs) [83] found that concurrent use of VEGFR-TKIs and factor Xa inhibitors (primarily LMWH) increases the risk of clinically significant bleeding events in patients with metastatic cancer (hazard ratio, HR 2.45; 95% CI, 1.28–4.69, *p* = 0.007). In addition, a retrospective study of 258 individuals did not detect a significant difference in their primary outcome, defined as rates of major bleeding in VEGF TKI plus anticoagulation compared to VEGF TKI without anticoagulation (8% vs 3%, *p* = 0.095). However, in this study a higher bleeding risk was noted in the composite endpoint of major and minor bleeding (OR, 2.73; 95% CI, 1.36–5.46; *p* = 0.005) [64]. This underpins the importance of nuanced clinical decision making in these at-risk groups.

Another retrospective study [84] of 100 patients with chronic myeloid leukemia (CML) found that those with TKI treatment (imatinib, dasatinib, nilotinib, bosutinib, or ponatinib) and selective serotonin reuptake inhibitors (SSRI) had a statistically significant higher incidence of critical site major bleeds compared to those without SSRIs. In addition, bleeding risk appears to depend on the specific TKI, as all observed bleeding events occurred in those with imatinib or dasatinib [84]. However, the risk of major bleeding or thrombotic events were similar. Previous studies reported substantial interindividual variability in the effects of different TKIs [85].

Current and emerging TKIs play a role in platelet activation and signalling pathways through off‐target effects [71]. These include pathways downstream of: (1) the immunoreceptor tyrosine-based activation motif (ITAM)-associated receptor glycoprotein VI (GPVI), (2) integrins such as αIIbβ3, (3) G protein-coupled receptors (GPCRs), and (4) the Janus kinase (JAK)/signal transducer and activator of transcription (STAT) signalling pathways [86]. However, the mechanism of BTK inhibitors on platelet function and haemostasis is unclear. Previous studies [87–89] suggested that GP1b and GPVI signalling pathways of the platelet receptors contributed potential bleeding events from ibrutinib. Patients who had mild bleeding do not require BTK inhibitor discontinuation [88], but if major bleeding occurs temporary cessation is advised [90]. To minimise the risks of perioperative bleeding, ibrutinib dose adjustment or urgent platelet transfusion should be considered [62, 88, 91].

### Management strategies for TKI-related thrombosis and bleeding

The balance of risk between thrombosis and bleeding in cancer patients on TKI therapy poses a significant clinical challenge for all practitioners involved in a patient’s cancer and cardiovascular care. This in turn means providers must look to prevent thrombosis, and at times treat thrombosis, whilst also considering risk reduction for major bleeding events and vice versa. Therefore a collaborative multidisciplinary approach is favoured, often involving medical and radiation oncologists, haematologists, cardiologists and primary care practitioners with a focus on individualised patient centric care [92].

#### Baseline risk assessment

Before TKI initiation the utilisation of guideline directed cardiovascular (CV) risk assessment is essential, which now must also include an assessment of bleeding risk [40]. Risk factors for thrombosis may include; history of coronary or peripheral artery disease, stroke, diabetes, smoking, hypertension, hyperlipidaemia, as well as prior venous thromboembolism or hypercoagulable conditions [40, 93]. For bleeding, consider any history of bleeding disorders, peptic ulcer disease, malignant metastases in crucial areas such as the bowel, liver or brain, use of non-steroidal anti-inflammatory drugs (NSAIDs), concurrent indications and use of anticoagulants, or severe thrombocytopenia [94]. This upfront assessment can guide preventative strategies and allow informed open discussion with patients about their individualised risk.

#### Modification of identified risk factors

Aggressive management of modifiable risk factors in patients receiving TKIs, especially those known to increase thrombosis, is a crucial step towards prevention. Patients on pro-thrombogenic TKIs should have optimal control of blood pressure, cholesterol and blood sugar, and they should be advised on smoking cessation and regular exercise [95]. Risk factor modification also extends to individual bleeding risk—for example, patients should be made aware and in cases avoid concomitant non-vital medications that can compound bleeding (NSAIDs, fish oil supplements, etc.), and ensure good blood pressure control to reduce the risk of haemorrhagic strokes.

#### Prophylactic antithrombotic therapy

Whether to use prophylactic antiplatelet or anticoagulant therapy in patients on TKIs is a nuanced decision. There is no one-size-fits-all guideline, and it must be individualized. Limited evidence already exists in the use of novel oral anticoagulant (NOACs) with low dose apixaban in select patients with high-risk cancer, with low-dose Apixaban demonstrating effective decrease the likelihood of venous thromboembolism despite the use TKIs [96]. Additionally, for high-risk patients receiving ponatinib, a preventive approach involving low-dose aspirin (100 mg/day) has been advised for individuals aged 60 years or older to mitigate the risk of arterial occlusive eventsv [97]. However, as suggested above the balance is complex and both prophylactic measures may elevate the potential for bleeding complications.

Another consideration is in patients taking the TKI ibrutinib who subsequently develop atrial fibrillation, guidelines suggest anticoagulation for stroke prevention should be instituted but typically a direct oral anticoagulant (DOAC) is preferred over warfarin in this scenario (warfarin has commonly been avoided in ibrutinib trials due to bleeding concerns) [98]. In fact, evidence suggests DOACs may be safer to combine with TKIs than vitamin K antagonists. A retrospective study found that among patients on VEGFR-TKIs who needed anticoagulation, those on DOACs had bleeding rates comparable to those on LMWH or warfarin [64]. Thus, if anticoagulation is necessary, DOACs are generally the first choice (provided no contraindications like gastrointestinal cancer bleeding risk or drug–drug interactions).

#### Patient education, monitoring and early detection

Educating patients about the signs and symptoms of both thrombosis and bleeding could be potentially lifesaving and cannot be overemphasized. Patients on TKIs need to know how to identify these complications, and to seek immediate care for symptoms of stroke or heart attack, as well as report any significant bleeding [93, 99]. They should be instructed on lifestyle modifications (using a soft toothbrush, an electric razor for those at risk of bleeding, avoiding high-risk activities such as heavy contact sports if platelets are low) [100]. Education should also cover medication interactions that could worsen bleeding, and informing all providers (including primary care practitioners and dentists) that they are on a TKI that affects bleeding/thrombosis.

Patients on TKIs should be regularly monitored for signs of thrombosis and bleeding. This includes periodic clinical assessment and appropriate investigations. This may include opportunistic review in clinic or at cancer treatment centres. Furthermore, a collaborative approach with the multidisciplinary team including involving nurse practitioners in these assessments will likely increase diagnostic yield and improve patient outcomes. Ideally monitoring for both thrombosis and bleeding should also extend beyond the hospital and treatment setting to also encompass the central role that primary care practitioners play in a patient’s cancer journey.

#### Managing a thrombotic event

If a patient develops a thrombosis whilst on a TKI, management typically involves treating the thrombosis as per standard treatment protocols (e.g., anticoagulation for venous thromboembolism (VTE), antiplatelet therapy for strokes and peripheral vascular disease and dual antiplatelet therapy and possible stenting for acute myocardial infarction (AMI)). In the acute setting, temporary interruption of the TKI is often advisable until the clinical situation and treatment plan is established [51]. For example, in AMI patients TKI may be withheld initially in discussion with the patients cancer care team given the TKI could complicate interventions if it causes platelet issues or the patient needs surgery. Following longer-term decisions will need to be made between the multiple specialists (Cardiologist, Oncologists, Haematologist) and the patient to determine the safety of TKI resumption and secondary preventative strategies. If the thrombosis is clearly attributed to the TKI, switching to an alternative cancer therapy with less thrombotic risk is prudent if feasible. In CML, for instance, a patient who suffers a serious arterial event on ponatinib might be transitioned to asciminib or back to dasatinib (balancing cancer control vs risk). If no alternative exists and the TKI is essential, then secondary prophylaxis with antithrombotic therapy may be instituted along with close monitoring [101]. In all cases, a tailored collaborative approach is key where there is a paucity of evidence. Studies in mice and case reports of patients experiencing arterial occlusive events while on ponatinib have explored the use of N-acetylcysteine and pioglitazone as potential interventions [102]. LMWHs have been the standard of care for treating arterial and venous thrombosis until the revolution of DOACs which in most cases are proven as effective as LMWH, however, this marginally increased the rate of major bleeding. This prevention strategy may not be applicable in patients with thrombocytopaenia due to the significant increase in major bleeding.

#### Managing a bleeding event

If significant bleeding occurs on a TKI, holding or stopping the TKI is usually the first step. Further supportive measures are often dictated by bleeding severity and reversibility. For mild bleeding (for example Grade 1–2 epistaxis), local measures and temporarily withholding the drug may suffice [51, 103]. Often the TKI can be resumed at the same dose once bleeding has resolved [51]. For more serious internal bleeding (for example an intracranial haemorrhage), the TKI should be stopped, and standard critical care for bleeding is required in discussion with appropriate oncological and haematological consultation [51, 103]. This may include blood product support (packed red blood cells, platelet transfusions if thrombocytopenic or suspected platelet dysfunction), cautious use of haemostatic medications (such as tranexamic acid), and urgent intervention (endoscopy, interventional radiology or surgery) as needed. Platelet transfusion is an important consideration for BTK inhibitor TKI treated patients with major haemorrhage given that ibrutinib causes irreversible BTK binding in existing platelets, transfusing new platelets can partially restore haemostatic function [91]. Of note, ibrutinib has a relatively short plasma half-life (~6 h), thus drug levels decline quickly and often withholding the drug and supporting the patient for a day or two can allow restoration of platelet function as new platelets are made [86, 91].

Decisions around resuming the TKI after a serious bleed are complex an often switching to alternatives therapies of a different class should be considered [91]. If the informed decision is made to restart a TKI, the specialists involved may decide on a dose-reduction if possible and ensure all reversible bleeding risk factors are also addressed [51].

### Coordination of care a collaborative balanced approach

As highlighted above, involvement of a multidisciplinary team is invaluable and allows for improved patient outcomes across the spectrum of cardio-oncology care [104]. The collaborative approach including the Cardiologist or Cardio-oncologist, Haematologist, Medical and Radiation Oncologists, Primary Care Practitioners along with the nursing and allied health teams allows for informed decision making between multiple specialised fields alongside the patient throughout their cancer journey [104]. Complex decisions around TKI treatment risks, prevention and management of possible complications such as thrombosis and bleeding should be shared when there is an absence of high-grade randomised data to guide clinical practice. This in-itself highlights the important need for further large-scale and quality international collaborative clinical trials in the area. With an ever-increasing plethora of TKI therapies becoming widely available in clinical use globally comes an equal need for close attention to adverse effects and treatment related complications. This can in many ways be better captured with improved access and input from multiple sites into global registries along with stringent reporting of adverse outcomes to overseeing authorities and government bodies approving therapies in their respective countries.

## Conclusion

TKIs have transformed the therapeutic landscape of many cancers, improving survival and outcomes. However, their use comes with the responsibility of managing unique toxicity profiles, notably the paradoxical risks of thrombosis and bleeding. This comprehensive scoping review highlights that certain TKIs can dramatically increase the risk of arterial thrombotic events, whereas others exert anti-platelet effects and may lead to bleeding. These effects stem from TKIs’ mechanisms of action at the molecular level—targeting kinases that, beyond their role in cancer, also play roles in vascular and platelet homeostasis. Clinicians must be vigilant in monitoring patients on TKIs for signs of clotting or haemorrhage, and be proactive in mitigating these risks through risk factor control, judicious use of concomitant medications, and prompt management of any events.

The variation across different TKIs in their thrombotic/bleeding profiles means therapy should be individualized. A “one-size-fits-all” approach does not apply; instead, understanding the pharmacology of each TKI guides safer use. The growing field of Cardio-Oncology faces significant challenges with complex areas of decision making for clinicians, such as these areas where conclusive randomized data for clinical scenarios are lacking. Large registries and post-marketing studies will assist in further clarifying the incidence and management approach of the rapidly evolving landscape of TKI therapies, and will help us all better understand TKI-associated thrombosis and bleeding. From a clinical perspective, close collaboration and a multidisciplinary approach between specialists, generalists and nursing and allied care givers is essential to navigate the delicate balance of preventing thrombosis without causing bleeding (and vice versa). For patients, education and open communication about symptoms can lead to early intervention and better outcomes.

TKIs encapsulate the progress of precision medicine in Oncology, and with that precision comes the need for precise management of side effects. By appreciating the mechanisms linking TKIs to thrombosis and bleeding, and by applying evidence-based management strategies, we can maximize the benefits of these drugs while minimizing their harms. Future research should focus on identifying predictive biomarkers for thrombotic or bleeding complications so that truly individualized preventive measures can be implemented. Moreover, novel TKIs with improved safety profiles will hopefully allow patients to receive the anticancer benefits without tipping the haemostatic balance. Until then, diligent monitoring and multidisciplinary care remain the cornerstones for managing the complex interplay of thrombosis and bleeding in patients on tyrosine kinase inhibitors.

## Data Availability

No datasets were generated or analysed during the current study.

## Abbreviations

ALK: Anaplastic lymphoma kinase
ALL: Acute lymphoblastic leukaemia
ATP: Adenosine triphosphate
AXL: Tyrosine-protein kinase receptor UFO
BCR-ABL: Philadelphia chromosome, breakpoint cluster region Abelson murine leukaemia 1
BRAF: V-Raf murine sarcoma viral oncogene homolog B
c-KIT: Proto-oncogene c-KIT/mast/stem cell growth factor receptor
BTK: Brutons tyrosine kinase
CML: Chronic myeloid leukaemia
DOAC: Direct oral anticoagulant
EGFR: Endothelial growth factor receptor
ErbB4: Receptor tyrosine-protein kinase erbB-4
FGFR: Fibroblast growth factor receptor
FLT3: Feline McDonough sarcoma like tyrosine kinase 3/Cluster of differentiation antigen 135
GP: Glycoprotein
GPCR: Glycoprotein coupled receptor
HER2: Receptor tyrosine-protein kinase erbB-2
HER2+: Receptor tyrosine-protein kinase erbB-2 expression/positivity
IL: Interleukin
JAK-STAT: Janus kinase
KIT: Proto-oncogene c-KIT/mast/stem cell growth factor receptor
LMWH: Low molecular weight heparin
MAP2K: See MEK
MEK: Mitogen-activated protein kinase kinase/MAP2K
MET: Hepatocyte growth factor receptor
NET: Neutrophil extracellular trap
NSAID: Non-steroidal anti-inflammatory drug
NSCLC: Non-small cell lung cancer
PAI-1: Plasminogen activator inhibitor-1
P13K: Phosphoinositide 3-kinases, involved in the PI3K/AKT/mTOR pathway
PDGFR: Platelet derived growth factor receptor
Ph+: Philadelphia chromosome positive
PMC: Platelet-leukocyte complex
RET: RET (rearranged during transfection) proto-oncogene
ROS1: Proto-oncogene tyrosine-protein kinase ROS
RTK: Receptor tyrosine kinases
SRC: Proto-oncogene tyrosine-protein kinase Src (sarcoma)
TAM: Tyro-3, Axl, and Mer family of tyrosine kinases
TKI: Tyrosine kinase inhibitor
VEGFR: Vascular endothelial growth factor receptor
VEGFI: Vascualar endothelial growth factor inhibitor
vWF: Von Willenbrand factor
